# Rhythmic dance interventions and dance/movement therapy for depression and anxiety: a structured narrative review of clinical evidence, candidate mechanisms, and implementation considerations

**DOI:** 10.3389/fpsyt.2026.1944858

**Published:** 2026-10-19

**Authors:** Xiaopeng Chen, Ting Wang, Jun Wang, Jing Tan

**Affiliations:** 1 School of Music and Dance, South-Central Minzu University, Wuhan, China; 2 Department of Pediatric Rehabilitation Medicine, Hubei Maternal and Child Health Hospital, Wuhan, China; 3 International Center for Redox Biology and Precision Medicine of Hubei Province, Hubei University of Technology, Wuhan, China; 4 Department of Basic Sciences, Wenhua College, Wuhan, China

**Keywords:** anxiety, dance intervention, dance/movement therapy, depression, psychological intervention, safety screening

## Abstract

**Background:**

This review synthesized evidence on rhythmic dance interventions (RDI) and dance/movement therapy (DMT) for depression and anxiety, clarified intervention boundaries, and proposed mechanism-informed and implementation-oriented recommendations.

**Methods:**

A structured narrative review was conducted using targeted literature searches of PubMed/MEDLINE, PsycINFO, Web of Science, Scopus, and the Cochrane Library from inception to 11 September 2026, supplemented by citation tracing. Sources were selected for relevance to clinical evidence, candidate mechanisms, intervention reporting, safety considerations, and implementation issues. The review was designed as a narrative and translational synthesis rather than a systematic review or meta-analysis.

**Results:**

Current evidence suggests that DMT and structured RDI may be associated with improvements in depressive symptoms, anxiety-related distress, stress, quality of life, and psychosocial functioning, although the evidence is heterogeneous and the most direct clinical evidence concerns DMT in adults with depression. Candidate mechanisms include rhythmic entrainment, behavioral activation, reward engagement, emotion regulation, interoceptive and autonomic regulation, social connectedness, and interpersonal co-regulation. However, direct mediation evidence is still insufficient, and key intervention features such as rhythm, synchrony, movement structure, interpersonal configuration, and reflective processing should be treated as reportable and potentially modifiable components rather than validated therapeutic dose parameters.

**Conclusions:**

RDI and DMT may represent promising complementary approaches for depression and anxiety, but they are not interchangeable and should not be regarded as substitutes for psychiatric, psychological, or medical care. Clearer intervention taxonomy, FITT + 3R reporting framework, mechanism-informed outcomes, adverse-event monitoring, and further evaluation of safety and implementation are needed to support responsible translation into healthcare and community settings.

## 1 Introduction

Depression and anxiety are common, disabling, and frequently co-occurring mental health conditions (1–3). They are associated with impaired academic and occupational functioning, reduced quality of life, interpersonal difficulties, and substantial personal and societal burden (1, 2, 4). Although they are diagnostically distinct, depression and anxiety share several transdiagnostic processes, including heightened negative affect, repetitive negative thinking, dysregulated arousal, reduced reward responsiveness, avoidance, and social withdrawal (5–7). These shared processes provide a rationale for interventions that target affective, bodily, motivational, and interpersonal regulation across diagnostic boundaries.

Evidence-based treatments, including structured psychotherapies and pharmacological interventions, benefit many individuals with depression and anxiety (2). However, real-world care remains limited by barriers to access, variable treatment response, residual symptoms, relapse, and difficulties with engagement and adherence (8). These limitations are especially relevant in community, school, university, rehabilitation, and low-resource settings, where scalable and acceptable adjunctive approaches may be valuable (7). Movement-based and embodied interventions may help address some of these gaps by engaging action, bodily awareness, affective expression, and social participation rather than relying exclusively on verbal cognitive processing (9, 10).

Rhythmic dance interventions (RDI) and dance/movement therapy (DMT) are two related but distinct embodied approaches (10, 11). RDI typically involves structured, music-accompanied movement delivered in group or class formats, emphasizing participation, enjoyment, and sustained engagement (11). These features make them amenable to implementation in schools, community programs, preventive health settings, and rehabilitation contexts (12). DMT, by contrast, is a psychotherapeutic modality delivered within a therapeutic relationship and guided by clinical aims (13). It uses movement experience, bodily awareness, nonverbal expression, interpersonal attunement, and reflective integration to support emotional, cognitive, social, and bodily integration (9, 13). Although RDI and DMT may share components such as movement, rhythm, synchrony, and group participation, they differ in clinical intent, facilitator training, therapeutic process, and safety responsibilities (9, 10). They should therefore not be treated as interchangeable interventions.

Existing reviews and meta-analyses suggest potential benefits of dance-based interventions and DMT for depressive symptoms, anxiety-related outcomes, quality of life, and psychosocial functioning (9–11). However, interpretation remains complicated by substantial variation in populations, intervention formats, facilitator qualifications, treatment duration and intensity, comparator conditions, outcome measures, and follow-up periods (14–16). Moreover, mechanistic interpretations remain preliminary. Rhythmic entrainment, behavioral activation and reward engagement, emotion regulation, interoceptive and autonomic regulation, social connectedness, and interpersonal co-regulation have all been proposed as candidate pathways (17–19), but few studies directly link a specific intervention component to a proximal process measure and subsequent clinical outcome. Accordingly, the current literature requires both clearer differentiation of intervention types and more cautious integration of clinical and mechanistic evidence.

Against this background, this structured narrative review has three primary aims: to distinguish RDI from DMT in terms of clinical intent, delivery, and therapeutic characteristics; to synthesize the current clinical evidence for depression- and anxiety-related outcomes while considering differences in evidence directness across populations and intervention contexts; and to organize candidate mechanisms and implementation considerations into a provisional framework for future research, reporting, and responsible translation.

## 2 Methods for literature identification and narrative synthesis

### 2.1 Review design

This article was designed as a structured narrative review and mechanism-informed translational synthesis, rather than a systematic review or meta-analysis. The aim was to integrate clinical evidence on RDI and DMT for depression and anxiety with relevant mechanistic and implementation literature, and to develop provisional considerations for intervention classification, reporting, safety, and future research.

A structured literature-identification and source-selection process was used to improve transparency and reproducibility. However, the review was not intended to identify an exhaustive corpus of all eligible studies, estimate pooled effect sizes, or provide a formal certainty-of-evidence grading. To improve the methodological interpretation of the primary clinical studies summarized in **Table 1**, design-appropriate risk-of-bias assessments were conducted as an adjunct to the narrative synthesis rather than as eligibility criteria or as part of a formal systematic-review evidence-grading process. Accordingly, the findings should be interpreted as an evidence-informed narrative synthesis and purposive evidence map rather than as a systematic estimate of intervention efficacy or evidence certainty.

**Table 1:** Clinical evidence map for RDI and DMT in depression, anxiety, and related psychosocial outcomes.

Source	Study design	Sample size (N)	Population/setting	Clinical status	Risk of bias	Intervention type	Comparator/context	Outcomes assessed	Direction of findings	Outcomes and measurement instruments	Main findings/statistical significance	Mechanism/implementation reporting	Key limitation
Ding et al., 2026 (20)	Evaluator-blinded parallel-group randomized controlled trial	N = 84	Rural left-behind youth with social anxiety	Symptom-defined/elevated symptoms	Some concerns (RoB 2)	Latin dance, mindfulness, or combined intervention	No-intervention control	Social anxiety	All interventions reduced social anxiety; combined intervention showed the most sustained effects	Social Anxiety Scale for Adolescents (SASA; Fear of Negative Evaluation and Social Avoidance subscales)	Significant group × time effect for social anxiety (F (6,192) =7.85, p<0.01); all intervention groups improved vs control (p<0.05), with the combined intervention showing the largest sustained reduction.	Components were compared, but mechanisms were not directly tested	Small sample; self-reported outcomes; limited generalizability
Nam et al., 2026 (21)	Quasi-experimental controlled study	N = 55	Female adolescents in a juvenile detention center	General/at-risk population	Serious risk (ROBINS-I)	DMT; 24 sessions over 8 weeks	Usual institutional routine/non-DMT control	Anxiety symptoms; mean body temperature; plasma dopamine	DMT was associated with reduced anxiety scores and increases in thermoregulatory and dopamine-related physiological indices	Beck Anxiety Inventory (BAI); mean body temperature; plasma dopamine by HPLC	BAI decreased by 16% (p<0.001); mean body temperature increased by 0.11 ± 0.07 °C (p<0.001); plasma dopamine increased by ~30% (p<0.001).	Included psychophysiological measures, but did not establish a complete mediation pathway	Single-center, all-female, quasi-experimental design; limited generalizability; preliminary mechanism inference
Zhao et al., 2025 (22)	Randomized controlled intervention study	N = 142	College students with depressed mood	Symptom-defined/elevated symptoms	Some concerns (RoB 2)	Modern dance, Chinese classical dance, jazz, and ballet interventions; 20 min/session, 4 times/week, 12 weeks	Non-dance control group	Self-rating depression scale; heart rate variability/autonomic nervous system indices	Dance interventions improved depressive mood and autonomic indices, with modern dance showing the strongest overall effect in this study	Zung Self-Rating Depression Scale (SDS); heart-rate variability (HRV: HR, SDNN, LF/HF and related indices)	SDS decreased after classical dance (p=0.029), ballet (p=0.008), and modern dance (p<0.001), but not jazz (p=0.241) or control (p=0.591); between-group change differed (F(4,95)=4.67, p=0.002).	Heart rate variability was included as a physiological process measure	College sample with depressed mood rather than diagnosed depression; relatively small groups; no formal mediation analysis
Li et al., 2025 (23)	Randomized controlled trial	N = 1,261	Left-behind children	General/at-risk population	Some concerns (RoB 2)	Structured moderate-intensity dance	Usual living conditions	Loneliness; self-esteem	Reduced loneliness and improved self-esteem	Children’s Loneliness Scale (CLS); Rosenberg Self-Esteem Scale (RSES)	Loneliness decreased (F(2,1258)=74.516, p<0.001) and self-esteem increased (F(2,1258)=73.64, p<0.001).	Intervention dose was well reported; mechanisms were not directly measured	Self-reported outcomes; inactive comparator; region-specific population
Li et al., 2025 (24)	Randomized controlled trial	N = 60	Left-behind children with social anxiety and low self-concept	Symptom-defined/elevated symptoms	Some concerns (RoB 2)	Structured dance intervention	Usual lifestyle	Social anxiety; self-concept	Reduced social anxiety and improved self-concept	Social Anxiety Scale for Children (SASC); Piers-Harris Child Self-Concept Scale (PHCSS)	SASC group × time interaction was significant (F(2,56)=17.131, p<0.001); social anxiety and self-concept outcomes improved overall (reported p<0.05), with gains in selected PHCSS domains.	Intervention structure was reported; mediators were not tested	Small sample; short follow-up; inactive comparator
Liu et al., 2025 (25)	Randomized two-group repeated-measures intervention study	N = 86 enrolled; N = 70 completed	Policewomen with elevated state or trait anxiety	Symptom-defined/elevated symptoms	High risk (RoB 2)	Mindfulness-based aerobic dance; 40 min/session, twice weekly, 12 weeks	Traditional aerobic dance	State anxiety; trait anxiety; emotion regulation	Mindfulness-based aerobic dance showed earlier and stronger improvements in anxiety and emotion regulation than traditional aerobic dance	Chinese Spielberger State-Trait Anxiety Inventory (STAI: SAI/TAI); Emotion Regulation Questionnaire (ERQ)	Mindfulness-dance group: SAI p=0.043 and TAI p=0.006 at week 8; SAI p=0.003 and TAI p<0.001 at week 12. Time × group interactions: TAI p=0.041 and cognitive reappraisal p=0.047.	Emotion regulation was assessed as a plausible process variable	No non-exercise usual-care control; self-report outcomes; attrition; component-specific effects remain uncertain
Christopher et al., 2025 (26)	Exploratory convergent mixed-method case series	N = 3	Adults with treatment-resistant depression	Diagnosed disorder	Methodological concerns (JBI case-series appraisal)	Group DMT	Uncontrolled clinical context	Stress; interoception; cortisol; embodied experience	Suggested improvements in self-regulation and embodied awareness	Perceived Stress Scale-10 (PSS-10); Multidimensional Assessment of Interoceptive Awareness-2 (MAIA-2); hair cortisol; interviews/focus groups	Integrated quantitative and qualitative findings suggested improved self-regulation, interoceptive awareness, and body-related experience; no inferential significance was reported for efficacy because of the very small case series.	Interoceptive, qualitative, and cortisol measures were included	Extremely small sample; no control group
Agres and Chen, 2025 (27)	Pilot parallel randomized controlled trial	N = 76	University students	General/non-clinical population	Some concerns (RoB 2)	Performing arts intervention including facilitated drumming and dancing; 10 weeks	Control group	Depression, anxiety, stress; quality of life; resilience; social networking; creativity; qualitative feedback	Intervention was associated with improvements in mental health, quality of life, and creativity; stress reduction was evident at follow-up	DASS-21; WHOQOL-BREF; Brief Resilient Coping Scale; Lubben Social Networking Scale; Experience of Creation Scale; B-MMR; qualitative feedback	Experimental-group DASS-21 stress improved at week 10 (p<0.05); selected WHOQOL domains and creativity also improved. Dance-specific effects cannot be isolated from the combined arts program.	Engagement and qualitative implementation experience were assessed	Combined drumming and dancing intervention; dance-specific effects cannot be isolated; university well-being sample rather than clinical depression/anxiety
Bleile et al., 2025 (28)	Quasi-experimental school-based controlled study	N = 549	Refugee children aged 10–15 years in Uganda	General/at-risk population	Serious risk (ROBINS-I)	TeamUp movement-based mental health promotion intervention	Care as usual/school-based comparison	Psychosocial well-being; friendships; attitude to school; traumatic stress; depressive symptoms; quality of life; physical health; social connectedness; agency	Reported improvements in psychosocial well-being, school-related outcomes, traumatic stress, quality of life, physical health, connectedness, and agency, but not consistently in depressive symptoms	SCWBS; MSLSS Friends/School; CRIES-8; KIDSCREEN-10/52; MAIA-2 Noticing; Affective Reactivity Index; Short Mood and Feelings Questionnaire; TeamUp mechanism scale	Psychosocial well-being p=0.01, school satisfaction p=0.004, traumatic stress p<0.001, HRQoL p=0.001, physical health p=0.014, social connectedness p=0.007, and agency p=0.005 improved; depressive symptoms were not significantly different.	Social connectedness and agency were assessed as implementation-relevant process domains	Movement-based rather than specifically dance or DMT; child refugee context; not directly focused on adult depression/anxiety
Benítez et al., 2025 (29)	Small-scale uncontrolled action-research study	N = 24 enrolled; N = 16 completed	Adults over 40 years old practicing Argentine tango	General/non-clinical population	Methodological concerns (JBI quasi-experimental appraisal)	Argentine tango dance	No non-dance control group	Functional fitness; blood pressure; quality of life; sleep; depressive symptoms	Functional capacity and systolic blood pressure improved, but quality of life, sleep, and depressive symptoms did not significantly change	WHOQOL-BREF; Pittsburgh Sleep Quality Index (PSQI); Patient Health Questionnaire-9 (PHQ-9); functional fitness tests; blood pressure	No significant change in quality of life, sleep, or depressive symptoms; 1-min sit-up improved (p=0.0081 and p=0.0005), 6-min step improved (p=0.0435), and systolic blood pressure decreased (p=0.0019).	Useful for functional and safety-related reporting; no mechanism testing	Small uncontrolled study; participants were already tango practitioners; limited relevance to depression/anxiety efficacy
de Souza et al., 2025 (30)	Randomized clinical trial with sequential dance interventions	N = 23	Women after breast cancer surgery	Other clinical population	Some concerns (RoB 2)	Free dance and dance therapy	Routine-activity control	Depression; anxiety; self-esteem; body image	Free dance improved depression; dance therapy improved anxiety	Self-Esteem Scale; Beck Anxiety Inventory (BAI); Body Image after Breast Cancer; Beck Depression Inventory (BDI)	Free dance improved depressive symptoms (within-group p=0.013; between-group p ≤ 0.001); dance therapy improved anxiety (p ≤ 0.001); body-image limitation domain differed (p=0.039).	Intervention dose and adherence were reported; mechanisms were not tested	Very small sample; differential adherence; complex intervention sequence
Volpe et al., 2025 (31)	Single-blind parallel-group pilot randomized controlled trial	N = 24	People with Parkinson’s disease	Other clinical population	Some concerns (RoB 2)	Multisensory artistic dance intervention	Conventional physiotherapy	Depression; anxiety; apathy; cognition; mobility	Favorable effects on selected emotional and cognitive outcomes	Beck Depression Inventory (BDI); STAI-I; Apathy Evaluation Scale (AES); motor and cognitive measures; adverse events	Greater percentage improvement with Dance Well for BDI (p=0.006), STAI-I (p<0.001), and AES (p<0.001); no Dance Well adverse events or dropouts.	Feasibility, adherence, and safety were reported	Small pilot sample; short intervention duration
Xu et al., 2024 (32)	Randomized controlled trial	N = 36	University students with depression and anxiety symptoms	Symptom-defined/elevated symptoms	Some concerns (RoB 2)	Intensive DMT	Wait-list control	Depression; anxiety	Reduced depression and anxiety, with maintained follow-up effects	Zung Self-Rating Depression Scale (SDS); Zung Self-Rating Anxiety Scale (SAS)	DMT reduced anxiety and depression vs wait-list immediately post-treatment and benefits were maintained at 3 months (reported significant between-group effects; p<0.05).	Therapeutic structure was described, but active components were not isolated	Small sample; wait-list comparator; intensive delivery format
He et al., 2022 (33)	Prospective assessor-blinded two-arm randomized controlled trial	N = 176	Women receiving chemotherapy for breast cancer	Other clinical population	Some concerns (RoB 2)	Culturally adapted dance intervention	General health consultation	Depression; fatigue; sleep disturbance; quality of life	Reduced depression and related symptom burden and improved quality of life	Brief Fatigue Inventory; Pittsburgh Sleep Quality Index; Patient Health Questionnaire-9 (PHQ-9); Functional Assessment of Cancer Therapy-Breast (FACT-B)	Dance reduced the fatigue–sleep disturbance–depression symptom cluster (p=0.003) and improved quality of life at week 17 (p=0.001); individual symptoms were also less severe.	Delivery format and implementation procedures were well reported	Limited blinding; variable home-practice fidelity; geographically restricted sample

This table is intended as a purposive clinical evidence map rather than a systematic review table. Outcome instruments and statistical significance are reported where available in the original publications; exact P values were not reconstructed when not explicitly reported. Risk-of-bias judgments are review-author assessments based on information available in the published reports and were used to contextualize interpretation rather than as eligibility criteria. Detailed domain-level assessments are provided in **Supplementary Figure S2**. Clinical status was standardized as diagnosed disorder, symptom-defined/elevated symptoms, other clinical population, or general/at-risk/non-clinical population. Findings should be interpreted in light of heterogeneity in study design, population, intervention format, comparator condition, and outcome assessment.

### 2.2 Search strategy and information sources

A structured literature search was conducted in PubMed/MEDLINE, PsycINFO, Web of Science, Scopus, and the Cochrane Library from database inception to 11 September 2026. Three complementary search strategies were conducted within each database to identify (1) clinical evidence, (2) candidate mechanistic evidence, and (3) implementation- and safety-related evidence. Each strategy combined a common dance/movement intervention block with domain-specific terms. This approach was used to avoid requiring clinical studies to contain mechanistic or implementation terminology. Search syntax was adapted to the indexing structure and search functions of each database. Complete database-specific search strategies, including search terms/descriptors, database-specific syntax, predefined limits or filters, and domain-specific retrieval counts, are provided in **Supplementary Table S1**. The literature-identification and evidence-selection process is summarized in **Supplementary Figure S1**. Because individual records could be retrieved by more than one search domain, domain-specific search yields were not summed to generate a single raw-record count.

### 2.3 Source selection criteria

Sources were considered for inclusion according to their relevance to one of three review domains: clinical evidence, candidate mechanisms, or healthcare implementation.

Clinical evidence sources included peer-reviewed primary intervention studies and evidence syntheses examining RDI, structured dance programs, DMT, or clearly defined DMT-informed or embodied movement interventions in relation to depression, anxiety, stress, affective symptoms, psychological well-being, quality of life, or psychosocial and functional outcomes. Within this domain, direct clinical evidence referred primarily to studies in populations with diagnosed depressive or anxiety disorders or explicitly defined depressive or anxiety symptoms, whereas studies in other clinical or non-clinical populations were treated as supportive or contextual evidence rather than equivalent direct evidence.

Mechanistic sources were included when they addressed processes plausibly relevant to dance-based interventions, including rhythmic entrainment, reward and motivation, behavioral activation, emotion regulation, interoception, autonomic regulation, interpersonal synchrony, social connectedness, and therapeutic processes. These sources informed candidate mechanisms and measurement targets but were not treated as direct clinical evidence unless they directly evaluated a dance-based or DMT intervention with relevant mental health outcomes.

Implementation-relevant sources were included when they informed intervention reporting, facilitator qualification, safety, referral procedures, adverse-event monitoring, acceptability, accessibility, fidelity, or healthcare implementation. These sources informed translational considerations rather than treatment-effect conclusions. Sources were excluded if they were not peer-reviewed, were unavailable in English, were conference abstracts, protocols, dissertations, or non-scholarly commentaries, lacked a clearly relevant dance-, movement-, mechanistic-, or implementation-related component, or did not provide sufficient methodological or conceptual information for the intended evidentiary role. Studies focused exclusively on motor or physical rehabilitation without relevant psychological, mechanistic, or implementation outcomes were also excluded.

### 2.4 Source selection and evidence-map construction

After cross-database deduplication, 8,835 unique records were available for relevance screening. Domain-specific database yields are presented in **Supplementary Table S1**, and the overall literature-identification and evidence-selection process is summarized in **Supplementary Figure S1**. Because the three search domains overlapped substantially, these search yields were not treated as mutually exclusive record counts. Titles and abstracts were screened independently by two authors (X.C. and J.T.) for relevance to the three predefined review domains, and potentially relevant publications were subsequently examined in greater detail. Disagreements regarding source relevance, intervention classification, or evidentiary role were resolved through discussion and consensus. Because source selection in this structured narrative review was purposive and iterative rather than based on a closed systematic-review eligibility pathway, full-text exclusions were not recorded as a separate formal screening stage. Thirty-nine sources identified through database searching were ultimately retained, and 39 additional sources were identified through citation tracing and targeted source identification, resulting in 78 unique sources contributing to the narrative synthesis. Of these, 36 informed the clinical evidence synthesis, 37 informed the candidate mechanism synthesis, and 23 informed implementation and safety considerations; these categories were not mutually exclusive. The Cochrane Library searches identified 593 records for clinical evidence, 325 for candidate mechanisms, and 438 for implementation/safety. Because full CENTRAL records could not be batch exported under the available institutional access, these search yields were reported separately and were not incorporated into the automated cross-database deduplication process.

**Table 1** was constructed as a purposive clinical evidence map rather than an exhaustive inventory of all identified studies. Studies were prioritized according to the directness of their relevance to depression or anxiety, clarity of intervention characterization, representation of distinct intervention formats and populations, methodological informativeness, and contribution to understanding clinical or implementation relevance. Recency alone was not sufficient for inclusion.

### 2.5 Data charting and intervention classification

Sources included in the narrative synthesis were charted using a structured framework aligned with the three review domains. Separate information fields were used for clinical, mechanistic, and implementation-relevant sources to preserve differences in evidentiary role. For clinical evidence sources, charted information included population, sample size when available, diagnostic or symptom characteristics, intervention type, facilitator qualification when reported, session frequency and duration, physical intensity when reported, comparator or delivery context, outcome domains and measurement instruments, follow-up duration, adherence, adverse events, principal findings, and reported statistical significance where available. Exact P values or other inferential statistics were recorded when explicitly reported in the original study and were not reconstructed when unavailable.

For mechanistic sources, charted information included the intervention or experimental component, candidate mechanism, mechanistic or process measure, clinical or behavioral outcome, and relevance to RDI or DMT. Mechanistic variables were treated as candidate measurement targets unless the original study directly tested temporal or mediating relationships between an intervention component, a proximal mechanism or process measure, and a clinical or functional outcome.

For implementation-relevant sources, charted information included reporting standards, facilitator requirements, delivery setting, acceptability, accessibility, adherence, fidelity, safety procedures, referral pathways, and adverse-event monitoring. These data were used to inform the reporting, implementation, and safety framework rather than to estimate treatment efficacy.

To reduce conceptual conflation, interventions were classified according to operational features rather than label alone. These features included clinical intent, facilitator credentials, degree of psychotherapeutic embeddedness, balance between structured and improvised movement, use of reflective processing, rhythm-related parameters, synchrony demands, interpersonal configuration, safety procedures, and referral capacity. Based on these features, interventions were categorized as structured RDI, therapeutic or clinical dance protocols, DMT-informed hybrid interventions, DMT, or related body psychotherapy or embodied movement approaches when appropriate.

Because this review was designed as a structured narrative review, data charting was used to support transparent comparison and conceptual synthesis rather than to generate a complete systematic-review database or formal quantitative evidence table. This separation was used to prevent mechanistic or implementation-relevant publications from being interpreted as equivalent to direct evidence of clinical effectiveness.

### 2.6 Risk-of-bias assessment, qualitative appraisal, and narrative synthesis

For the primary clinical studies summarized in **Table 1**, risk of bias was assessed using design-appropriate validated tools. Randomized controlled trials were assessed using the Cochrane Risk of Bias 2 (RoB 2) tool, non-randomized intervention studies using ROBINS-I, and case-series evidence using the relevant Joanna Briggs Institute (JBI) critical appraisal checklist, where applicable. Risk-of-bias judgments were used to support interpretation of the clinical evidence and were not applied as formal eligibility criteria. Summary judgments are presented in **Table 1**, with domain-level assessments provided in **Supplementary Figure S2**.

Evidence was appraised qualitatively according to relevance, methodological informativeness, and translational value. Clinical studies were considered more informative when they included randomized or controlled designs, clearly defined participant characteristics, adequately described intervention components, active or credible comparators, validated depression or anxiety measures, follow-up assessment, and reporting of adherence, fidelity, or adverse events. This qualitative appraisal complemented, but was kept conceptually separate from, the design-specific risk-of-bias assessment. It was used to guide interpretation within the narrative synthesis rather than to generate a formal certainty-of-evidence grade.

Mechanistic evidence was considered more informative when studies linked a reportable and potentially modifiable intervention component to a proximal mechanistic or process measure and then to a clinical or functional outcome. Mechanistic claims were intentionally framed as candidate pathways unless direct evidence from dance or DMT trials was available. Particular attention was given to evidence gaps, including the scarcity of mediation analyses, inconsistent reporting of rhythm and synchrony parameters, underreporting of adverse events, limited follow-up, and the limited number of trials simultaneously measuring intervention components, proximal process measures, and clinical or functional outcomes.

Implementation evidence was considered more relevant when studies reported feasibility, acceptability, adherence, equity, delivery setting, facilitator requirements, safety monitoring, adverse events, or referral procedures. These implementation-related findings were integrated with the clinical and mechanistic literature to inform the proposed taxonomy, FITT + 3R reporting framework, safety considerations, and implementation considerations.

Evidence was synthesized narratively according to both review domain and evidentiary directness. Clinical evidence was first considered according to whether it represented direct evidence in depression or anxiety, supportive evidence from other clinical populations, or broader contextual evidence. Candidate mechanisms were then synthesized separately, with explicit distinction between direct dance- or DMT-based findings and indirect evidence from adjacent fields. Implementation evidence was synthesized as contextual support for the proposed reporting, safety, and implementation considerations rather than as evidence of treatment efficacy.

No formal quantitative weighting or certainty-of-evidence grade was assigned. Risk-of-bias judgments were used descriptively to contextualize the primary clinical evidence rather than to generate a composite numerical quality score. Interpretive emphasis was additionally placed on study design, population relevance, intervention specificity, outcome validity, comparator quality, follow-up, and the directness of evidence supporting each conclusion.

## 3 Definitions and intervention taxonomy

### 3.1 Defining RDI and DMT

Dance-based interventions for depression and anxiety are often grouped together, but they differ substantially in purpose, delivery, training requirements, and proposed mechanisms, implementation requirements, and safety responsibilities (12, 34). For the purposes of this review, the term RDI was used to refer to structured or semi-structured movement programs in which music, rhythm, repeated movement phrases, and group participation were used to promote psychological well-being, affect regulation, physical activation, and social engagement. These interventions were often delivered in community, educational, preventive, rehabilitation, or adjunctive clinical settings. They might be led by dance instructors, exercise professionals, rehabilitation staff, or trained facilitators, and their therapeutic value was generally expected to arise from participation, movement activation, rhythmic cueing, enjoyment, mastery, and social connection (10, 11).

DMT, by contrast, referred to a psychotherapy-oriented intervention in which movement was used as a primary medium for emotional, cognitive, social, and bodily integration (11). DMT was typically delivered by a qualified dance/movement therapist or a clinician with relevant training and supervision. It involved clinical formulation, therapeutic attunement, movement observation, embodied expression, symbolic or relational exploration, and verbal or nonverbal integration of movement experience (13). Whereas RDI usually emphasized reproducible activity structures and scalable delivery, DMT emphasized individualized process, therapeutic relationship, clinical containment, and meaning-making through movement (17, 18).

These categories are related but not interchangeable. They may share movement, rhythm, music, group participation, and synchrony, but they differ in the role assigned to movement (10). In RDI, movement is usually the structured activity through which activation, engagement, and social participation occur (12). In DMT, movement is also a therapeutic language through which affect, relational patterns, bodily experience, and meaning are assessed and transformed (12, 35). This distinction is important for evidence synthesis because combining all dance-related approaches under a single label can obscure differences in target population, mechanism, facilitator responsibility, and safety requirements.

### 3.2 An operational taxonomy for classifying dance-based interventions

Because intervention labels are used inconsistently across the literature, classification should be based on operational features rather than name alone. Building on distinctions in the DMT, dance intervention, and intervention-reporting literature (10, 12), we proposed that dance-based interventions can be described along six dimensions: clinical intent, facilitator qualification, movement structure, reflective or therapeutic processing, interpersonal configuration, and safety and referral responsibility.

Clinical intent refers to whether the intervention is designed primarily for health promotion, prevention, adjunctive symptom reduction, rehabilitation, or psychotherapy (10, 36), whereas facilitator qualification refers to whether sessions are delivered by a dance instructor, exercise professional, rehabilitation provider, dance/movement therapist, psychotherapist, or multidisciplinary team (10, 37). Besides, movement structure describes the relative balance between structured movement sequences, semi-structured tasks, and improvisation (12). Reflective or therapeutic processing refers to whether the session includes no reflection, brief supportive discussion, structured emotional processing, or formal psychotherapeutic integration (9, 13). Moreover, interpersonal configuration describes whether the intervention is individual, dyadic, small-group, whole-group, synchronous, mirrored, partner-based, or primarily solo (13, 38, 39). Finally, safety and referral responsibility refers to the level of screening, monitoring, containment, adaptation, and referral capacity required for the target population (40, 41). Operational classification was based on the combined pattern of these six dimensions rather than on any single feature. Structured RDI was characterized primarily by reproducible rhythmic movement, health-promotion or adjunctive aims, limited psychotherapeutic processing, and delivery by dance, exercise, rehabilitation, or trained community facilitators. Therapeutic or clinical dance protocols retained a structured movement format but were adapted for a defined clinical population and generally involved greater clinical oversight without constituting formal psychotherapy. DMT was defined by explicit psychotherapeutic intent, delivery by an appropriately qualified dance/movement therapist or clinician, use of movement as a therapeutic medium, individualized or relational formulation, and integration of reflective or psychotherapeutic processing. Interventions combining structured rhythmic movement with selected DMT-derived therapeutic elements but not fully meeting these criteria were classified as DMT-informed hybrid interventions. Classification therefore reflected the overall configuration of clinical intent, facilitator qualification, movement structure, therapeutic processing, interpersonal configuration, and safety/referral responsibility.

Using this operational framework, dance-based interventions were organized into four pragmatic categories: structured RDI, therapeutic or clinical dance protocols, DMT-informed hybrid interventions, and DMT (**Figure 1**). Structured RDI include reproducible dance- or rhythm-based programs delivered primarily for health promotion, prevention, or adjunctive psychosocial support (10, 11, 14). Therapeutic or clinical dance protocols involve structured dance programs adapted for clinical populations but generally do not include formal psychotherapeutic processing (14, 42, 43). DMT-informed hybrid interventions represent a pragmatic category proposed in this review for programs that combine structured rhythmic movement with selected therapeutic elements, such as safety framing, guided reflection, co-regulation, or body-awareness practices (10, 44). This term is used here as a review-specific working category and should not be interpreted as an established professional or disciplinary classification. DMT embeds movement within a psychotherapeutic relationship guided by clinical formulation and therapeutic processes (10, 15, 45). Related body psychotherapy or embodied movement approaches may overlap with DMT but should be identified separately when they do not explicitly meet DMT criteria (46, 47).

**Figure 1:**
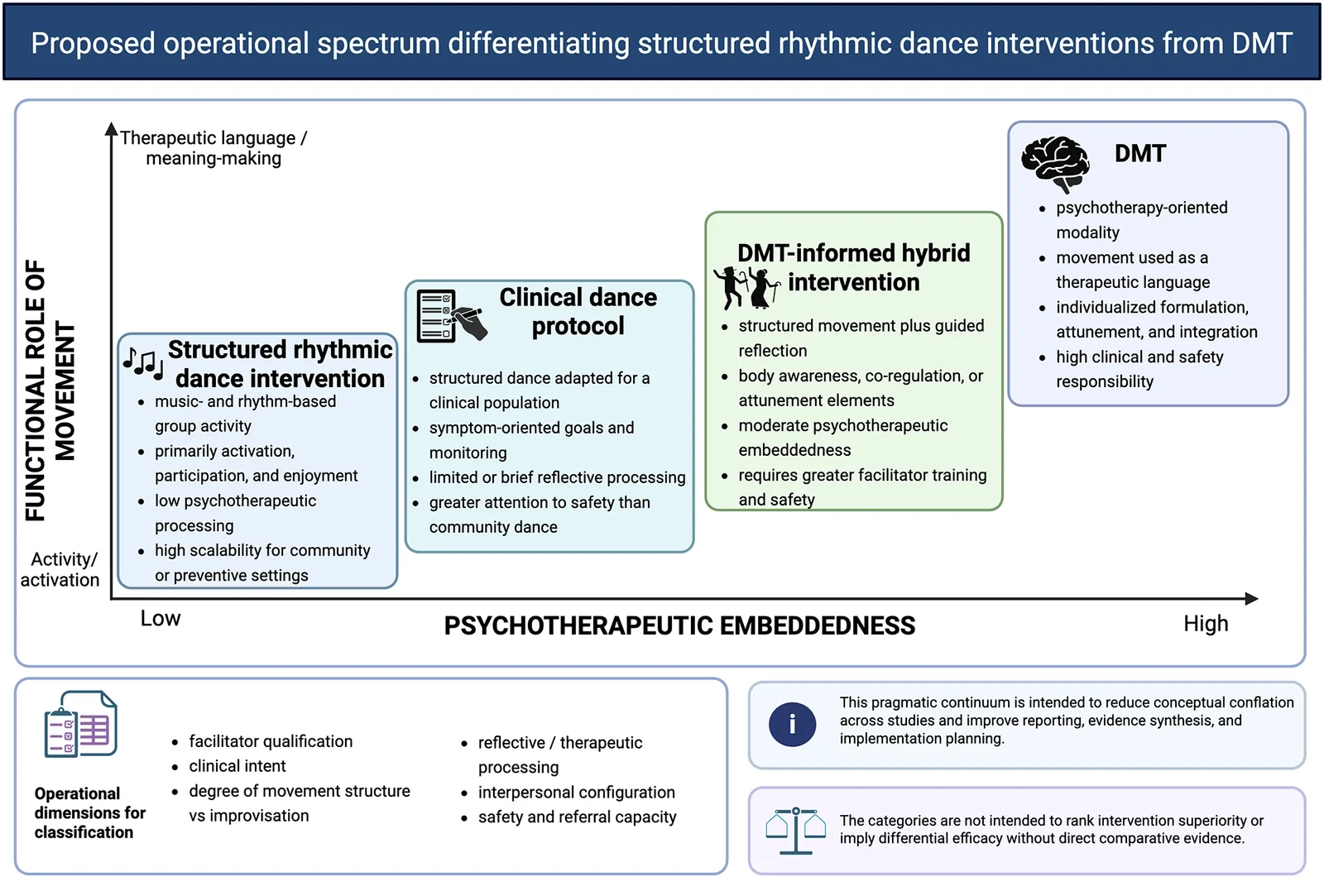
Proposed operational spectrum differentiating structured rhythmic dance interventions from DMT. The image illustrates a review-specific operational continuum ranging from structured rhythmic dance interventions to psychotherapy-oriented DMT, with clinical dance protocols and DMT-informed hybrid interventions positioned between these poles according to psychotherapeutic embeddedness and the functional role of movement. Classification is based on facilitator qualification, clinical intent, degree of movement structure versus improvisation, reflective or therapeutic processing, interpersonal configuration, and safety or referral capacity. The term “DMT-informed hybrid intervention” is used here as a review-specific working category rather than an established professional classification. The continuum is intended to reduce conceptual conflation across studies and support clearer reporting and evidence synthesis; it should not be interpreted as a validated treatment-matching framework, nor do the categories imply differential efficacy or intervention superiority in the absence of direct comparative evidence.

This taxonomy is not intended to rank interventions or imply that one format is universally superior. Instead, it is intended to improve reporting, reduce conceptual conflation, and clarify which evidence supports which type of intervention. Scalable rhythmic dance programs may be considered for well-being promotion, prevention, mild symptom presentations, or community implementation (9, 11, 14), whereas DMT or DMT-informed formats may be more appropriate when individualized therapeutic containment, trauma-informed pacing, or relational processing is required (9, 42). However, such matching recommendations should be treated as translational hypotheses unless tested directly in comparative or stratified trials. This taxonomy provides the conceptual basis for **Tables 1**–**4**, which summarize the clinical evidence, candidate mechanisms, implementation considerations, and operational distinctions among the four intervention categories, respectively.

**Table 2:** Candidate mechanisms, feasible measures, and implementation implications for dance-based mental health interventions.

Candidate mechanism	Relevant symptom or implementation target	Modifiable intervention components	Feasible measures	Evidence basis	Implementation implication
Rhythmic entrainment and structured attention	Action initiation, attentional anchoring, predictability, psychomotor slowing, anxiety-related uncertainty	Tempo, rhythmic regularity, beat salience, cueing modality, movement complexity, progression	Beat synchronization accuracy, movement persistence, perceived predictability, attentional task performance, adherence	Limited direct clinical evidence; primarily indirect evidence from rhythm, auditory beat perception, and sensorimotor synchronization research	Rhythm may be reported as a candidate modifiable component rather than treated as a validated therapeutic dose parameter
Behavioral activation and reward engagement	Anhedonia, low motivation, fatigue, reduced pleasure, behavioral withdrawal, poor engagement	Enjoyable or culturally meaningful music, progressive mastery, achievable movement tasks, supportive group participation	Positive affect, behavioral activation, self-efficacy, enjoyment, reward responsiveness, attendance, continuation of activity	Limited direct clinical evidence; additional indirect evidence from behavioral activation, physical activity, and reward-processing research	These components may inform hypotheses about activation and engagement, but their mediating role requires direct testing
Emotion regulation and reduction of repetitive negative thinking	Rumination, worry, emotional inhibition, avoidance, affective rigidity	Structured movement, guided improvisation, mirroring, expressive movement, reflective processing	Rumination, worry, emotion-regulation scales, affect ratings, ecological momentary assessment, session-level expression or avoidance ratings	Limited direct clinical evidence; indirect evidence from emotion-regulation and psychotherapy-process research	Particularly relevant as a candidate process in DMT and DMT-informed formats involving emotional expression and reflective processing
Interoceptive and autonomic regulation	Hyperarousal, somatic anxiety, panic-like sensations, fatigue, sleep disturbance, bodily disconnection	Breath-movement coupling, grounding, tempo modulation, graded intensity, predictable structure, arousal titration	Heart rate variability, respiration rate, perceived arousal, somatic anxiety, interoceptive awareness, body awareness, sleep quality, fatigue	Limited direct clinical evidence; primarily indirect evidence from interoception, autonomic regulation, stress physiology, and physical-activity research	Physiological indices should be treated as candidate process markers rather than mediators unless their relationship with clinical improvement is tested directly
Social connectedness and interpersonal co-regulation	Loneliness, social withdrawal, interpersonal avoidance, low belonging, reduced community participation	Group unison, circle formation, call-and-response, optional partner work, mirroring, therapist or facilitator attunement	Loneliness, social connectedness, group cohesion, therapeutic alliance, perceived safety, interpersonal functioning, qualitative feedback	Limited direct clinical evidence; indirect evidence from interpersonal synchrony and psychotherapy-process research	Group and interpersonal features may be relevant to affiliation and co-regulation, but objective synchrony should not be assumed to represent therapeutic benefit

Evidence-basis descriptions indicate the directness and source of support rather than a graded hierarchy of mechanistic certainty. “Direct clinical evidence” refers to dance-, RDI-, or DMT-based studies assessing candidate process variables alongside relevant mental health outcomes, whereas “indirect evidence” refers to findings from adjacent research fields. These mechanisms should be interpreted as candidate pathways and measurement targets rather than confirmed mediators of therapeutic change. Safety screening, referral thresholds, trauma-informed delivery, engagement, acceptability, adherence, and adverse-event reporting are addressed separately in **Table 3**.

**Table 3:** Proposed implementation and reporting considerations for dance-based interventions.

Domain	Proposed consideration or reporting item	Proposed practical consideration	Healthcare relevance
Intervention classification	Specify whether the program is structured rhythmic dance, clinical dance protocol, DMT-informed intervention, body psychotherapy, or DMT	Intervention classification may consider clinical intent, facilitator qualification, movement structure, psychotherapeutic processing, and safety responsibility rather than relying on the intervention label alone	Reduces conceptual conflation and clarifies scope of practice
Target population and clinical complexity	Report age, diagnosis or symptom profile, symptom severity, comorbidity, functional impairment, physical limitations, participant preference, trauma-related considerations when relevant, and delivery setting	Relevant considerations may include participant characteristics, functional needs, clinical complexity, facilitator qualification, delivery context, and available safety or referral capacity when selecting the intervention format	Supports appropriate alignment between intervention format, participant needs, facilitator responsibility, and clinical support requirements
Facilitator qualification and supervision	Report facilitator background, dance/exercise/DMT/psychotherapy training, clinical supervision, and referral responsibility	Community programs may be led by trained instructors, whereas clinically complex groups may warrant involvement of DMT therapists, mental health professionals, or multidisciplinary support	Clarifies safety responsibility, professional boundaries, and escalation capacity
FITT parameters	Report frequency, intensity, time, and type, including sessions per week, total program duration, session length, physical exertion, progression, dance style, and delivery mode	Potential risk-mitigation approaches may include graded intensity, warm-up and cool-down periods, low-impact options, seated alternatives, and predefined stop criteria	Enables replication while reducing risk for older adults, rehabilitation groups, and participants with physical limitations
Dance-specific components	Report music or cueing source, approximate tempo range when available, rhythmic regularity, cueing modality, movement complexity, synchrony demand, interpersonal configuration, and degree of improvisation	Rhythm and synchrony may be described as reportable and potentially modifiable components rather than as validated therapeutic dose parameters	Supports future component-level testing and avoids overstating rhythm-based prescription
Reflective or therapeutic processing	Report whether sessions include no reflection, brief supportive discussion, structured reflection, embodied emotional processing, or formal psychotherapeutic integration	For DMT and DMT-informed formats, reporting may specify how movement experience is integrated verbally or nonverbally	Distinguishes activity-based dance, supportive group work, DMT-informed formats, and psychotherapy-oriented DMT
Psychological safety and trauma-informed delivery	Potential screening considerations may include severe distress, trauma destabilization, dissociation, panic vulnerability, body-image distress, and social-evaluative sensitivity	Trauma-informed delivery may incorporate consent, choice, predictability, opt-out options, non-performance framing, bodily autonomy, and careful titration of touch, visibility, proximity, partner work, and improvisation	Supports safe participation among trauma-exposed, anxious, or clinically vulnerable participants
Referral thresholds and medical safety	Programs may predefine referral or exclusion considerations for suicidal ideation involving intent or plan, recent self-harm with acute safety concerns, mania/hypomania, psychosis, severe dissociation, severe panic symptoms, rapid symptom deterioration, unstable eating disorder symptoms, unstable cardiovascular disease, acute injury, severe pain, or high fall risk	Where relevant to the setting and population, programs may establish mental health and medical referral pathways before delivery	Helps reduce the risk of inappropriate substitution for indicated psychiatric, psychological, or medical care
Outcome, mechanism, and adverse-event monitoring	Studies and programs are encouraged to report validated symptom measures, functional outcomes, attendance, dropout, satisfaction, follow-up, candidate mechanisms, and adverse events	Potential outcomes and monitoring domains include depression, anxiety, quality of life, functioning, social connectedness, adherence, heart rate variability or interoceptive awareness when feasible, and both physical and psychological adverse events	Strengthens clinical interpretability, mechanism testing, and safety evidence
Implementation context, equity, and delivery mode	Report setting, cost, accessibility, cultural relevance, disability accommodations, privacy, digital access, and online/hybrid procedures	Delivery mode may be aligned with clinical complexity; for online or hybrid formats, relevant reporting considerations include platform, latency, music delivery, camera use, privacy, safety monitoring, and emergency contact procedures	Supports scalable, equitable, and sustainable implementation across community, school, primary care, rehabilitation, mental health, and digital settings

These proposed considerations are intended as a reporting and implementation aid rather than a validated checklist, clinical guideline, treatment prescription, or decision rule. They should be adapted to local professional standards, participant needs, facilitator qualifications, and available clinical or referral resources.

**Table 4:** Operational comparison of dance-based intervention categories.

Feature	Structured RDI	Therapeutic/clinical dance protocol	DMT-informed hybrid intervention	DMT
Primary clinical intent	Health promotion, prevention, or adjunctive psychosocial support	Symptom support or rehabilitation in a defined clinical population	Combined activity-based and therapeutic aims	Psychotherapy
Typical facilitator	Dance instructor, exercise professional, rehabilitation staff, or trained facilitator	Clinically supervised dance, rehabilitation, or allied-health professional	Trained facilitator or clinician with selected therapeutic competencies	Qualified dance/movement therapist or appropriately trained clinician
Movement structure	Mainly structured and reproducible	Predominantly structured, with clinical adaptation	Structured movement combined with selected exploratory or improvisational elements	Flexible, individualized, and often exploratory or improvisational
Role of movement	Primary activity through which activation, engagement, and participation occur	Clinically adapted therapeutic activity	Activity combined with selected therapeutic meaning-making or regulation processes	Primary therapeutic medium within a psychotherapeutic process
Reflective/therapeutic processing	Minimal or absent	Limited or supportive	Present but selective or partial	Integral to the intervention
Interpersonal configuration	Often group-based, synchronous, or instructor-led	Individual or group, depending on clinical context	Individual, dyadic, or group-based	Individual, dyadic, or group-based according to therapeutic formulation
Psychotherapeutic formulation	Not required	Usually absent or limited	May be partially incorporated	Explicitly incorporated
Safety and referral responsibility	Basic screening, adaptation, and escalation procedures	Greater clinical monitoring and referral capacity	Enhanced monitoring, containment, and referral procedures	Integrated clinical formulation, containment, monitoring, and referral responsibility
Typical setting	Community, school, wellness, rehabilitation, or adjunctive healthcare settings	Clinical or rehabilitation settings	Clinical, community, or hybrid settings	Mental health or other clinically supervised settings
Classification status	Pragmatic intervention category	Pragmatic clinical category	Review-specific proposed working category	Established professional therapeutic modality

The term “DMT-informed hybrid intervention” is used in this review as a pragmatic, review-specific working category and should not be interpreted as an established professional or disciplinary classification. Classification is based on the overall configuration of clinical intent, facilitator qualification, movement structure, reflective or therapeutic processing, interpersonal configuration, and safety/referral responsibility rather than on any single feature.

### 3.3 Candidate reportable components: rhythm, movement structure, and interpersonal configuration

A major advantage of dance-based interventions is that several components can be described and, in principle, manipulated (12, 37). These include tempo, beat salience, rhythmic regularity, cueing modality, movement complexity, synchrony demand, spatial organization, group size, and the prescribed-to-improvised movement ratio (12, 17, 18, 38). However, current clinical trials rarely reported these components in sufficient detail, and few studies tested whether specific rhythmic or interpersonal parameters mediate changes in depression or anxiety (10, 11). Therefore, these elements should currently be described as candidate reportable components rather than established therapeutic dose parameters.

For RDI, minimum reporting should include music or cueing source, approximate tempo range when available, rhythmic regularity, session structure, movement intensity, progression, synchrony requirements, and whether participants move individually, in pairs, or as a group (48, 49). For DMT and DMT-informed interventions, reporting should additionally include facilitator credentials, therapeutic orientation, use of mirroring or attunement, degree of improvisation, reflective or verbal processing, trauma-informed adaptations, and referral procedures. These details are necessary because the same surface label, such as “dance therapy” or “movement group,” may refer to very different interventions (50–52).

This operational approach also supports mechanism-informed research. Rather than asking whether dance works as a single undifferentiated intervention, future studies could test whether specific components, such as predictable rhythm, graded synchrony, guided improvisation, or therapist attunement, are associated with proximal changes in arousal regulation, reward responsiveness, emotion regulation, interoception, social connectedness, or therapeutic alliance. This distinction underpins the component–mechanism framework in **Table 2** and the reporting and safety checklist in **Table 3**.

## 4 Clinical evidence

### 4.1 Overview of the clinical evidence base and evidence hierarchy

**Table 1** summarizes the clinical evidence most relevant to depression, anxiety-related distress, stress, and broader functional and psychosocial outcomes. Overall, the current literature suggests that both DMT and RDI might improve psychological outcomes, although the strength, specificity, and generalizability of the evidence vary across intervention types and populations (15, 50, 53–56). The evidence base is highly heterogeneous. Existing studies differ substantially in diagnostic status, symptom severity, intervention format, facilitator qualification, comparator condition, session frequency and duration, physical intensity, movement structure, outcome selection, and follow-up duration (46, 57, 58). Accordingly, the literature should be interpreted as a heterogeneous body of evidence rather than as a uniform set of trials evaluating equivalent clinical populations or interventions.

For the purposes of interpretation, the evidence was organized according to evidentiary directness. Direct clinical evidence included studies conducted in participants with diagnosed depressive or anxiety disorders or with explicitly defined depressive or anxiety symptoms. Within this category, studies involving formally diagnosed disorders were distinguished from those involving elevated, subclinical, or symptom-defined presentations. Supportive clinical evidence referred to studies conducted in populations with other primary medical or neurological conditions, such as cancer or Parkinson’s disease, in which depression, anxiety, or related psychological outcomes were assessed. Broader contextual evidence included community, university, school, refugee, or general well-being samples, as well as broader movement-based interventions whose relevance to depression and anxiety was indirect. These categories were used to avoid treating heterogeneous populations and intervention contexts as equivalent forms of clinical evidence and to indicate evidentiary directness rather than a formal hierarchy of certainty, methodological quality, or risk of bias.

An additional distinction concerns intervention type. DMT and RDI should not be treated as interchangeable (10). DMT studies generally evaluate psychotherapy-oriented interventions and often involve clinically symptomatic populations, whereas RDI studies span a wider range of preventive, community, educational, rehabilitation, and adjunctive contexts. Meta-analytic findings have also suggested differences in effect patterns, with DMT effects appearing smaller but more homogeneous and dance intervention effects larger but more heterogeneous in some analyses (59). These differences reinforce the importance of interpreting findings according to both evidence directness and intervention type.

Within this evidence hierarchy, the most direct evidence for DMT concerns adult depression, whereas the RDI literature spans a broader but more heterogeneous range of depressive symptoms, anxiety-related distress, stress, and psychosocial outcomes (9, 16, 60, 61). The following sections therefore distinguish direct evidence from supportive and contextual evidence before comparing the clinical profiles of DMT and RDI.

### 4.2 Direct evidence for depression and anxiety: diagnosed disorders and symptom-defined populations

The most direct clinical evidence comprises studies involving participants with diagnosed depressive or anxiety disorders and studies recruiting participants on the basis of explicitly defined depressive or anxiety symptoms. These forms of evidence are both directly relevant to the focus of this review but should not be treated as equivalent. Studies conducted in diagnostically defined populations provide the closest evidence for the treatment of depressive or anxiety disorders, whereas symptom-defined studies provide evidence for individuals with elevated, subclinical, or otherwise clinically relevant symptoms without necessarily establishing a formal diagnosis.

Among diagnostically defined populations, the most direct evidence for DMT concerns adult depression. Systematic reviews and controlled studies have suggested that DMT may reduce depressive symptoms and improve psychological functioning in adults with depression and related internalizing difficulties (56, 62, 63). From a clinical perspective, DMT may be particularly relevant when depressive symptoms are accompanied by bodily disconnection, reduced agency, social withdrawal, emotional inhibition, or difficulty accessing experience through verbal reflection alone (22, 62). More recent controlled studies have also supported the potential value of group-based DMT as an adjunct to usual care, although the number of rigorous trials remains limited. DMT differs from structured dance programs in that movement is embedded within a psychotherapeutic process. Clinical studies commonly incorporate embodied expression, movement exploration, therapeutic attunement, and reflective integration, although the extent to which these elements contribute independently to treatment outcomes remains uncertain. Current evidence therefore supports DMT as a promising psychotherapy-oriented approach while remaining insufficient to determine which specific therapeutic components are responsible for improvement.

A second body of direct evidence concerns symptom-defined populations without a confirmed formal diagnosis. These studies include participants selected on the basis of elevated depressive symptoms, depressed mood, anxiety scores, social anxiety, or related psychological distress. Within this literature, structured RDI has been evaluated more frequently than formal DMT and has shown potential benefits for depressive and anxiety-related symptoms across university, youth, occupational, and other populations (9, 16, 60, 61). These findings are clinically relevant but should not be interpreted as equivalent to evidence from diagnostically defined depressive or anxiety disorders. In particular, improvement in symptom scores among participants with elevated distress does not establish efficacy for the treatment of a formally diagnosed disorder.

Important methodological limitations remain across both diagnostically defined and symptom-defined studies. Many DMT and RDI trials have small samples, heterogeneous protocols, variable comparator conditions, and limited follow-up. Active or credible comparators remain uncommon, making it difficult to separate intervention-specific effects from the effects of therapeutic attention, group participation, physical activity, expectancy, or general engagement (14, 64, 65). In addition, clinically relevant processes such as body awareness, emotional regulation, therapeutic alliance, and interpersonal attunement are inconsistently assessed and rarely tested as mediators (49, 63, 66). Taken together, the available evidence supports cautious optimism regarding dance-based interventions for depression- and anxiety-related outcomes, while the most direct psychotherapy-oriented evidence remains concentrated in DMT for adult depression. However, the evidence is not yet sufficient to establish superiority over other active interventions, identify reliable patient-level predictors of response, or determine which therapeutic components account for clinical benefit.

### 4.3 Supportive clinical and broader contextual evidence

Beyond studies directly targeting depressive or anxiety disorders or symptom-defined populations, dance-based interventions have also been evaluated in a range of other clinical contexts. Studies involving populations with cancer, Parkinson’s disease, and other medical or neurological conditions have reported changes in depressive or anxiety symptoms, quality of life, psychological distress, and related psychosocial outcomes (49, 67–69). These findings extend the potential relevance of dance-based interventions to medically complex populations and suggest that psychological benefits may accompany rehabilitation or supportive care. However, because depression or anxiety was not necessarily the primary diagnosis or treatment target, these studies should be interpreted as supportive rather than disorder-specific evidence.

Broader contextual evidence is derived from university, school, community, refugee, and general well-being samples, as well as from interventions in which dance is embedded within broader performing-arts or movement-based programs (28, 62, 70, 71). Such studies provide useful information on psychological distress, social connectedness, engagement, acceptability, quality of life, and functional or psychosocial outcomes, and may be particularly informative for understanding feasibility and implementation outside specialist mental health settings. Their relevance to the treatment of depression and anxiety remains indirect, however, when participants were not selected on the basis of a defined depressive or anxiety disorder or symptom threshold.

RDI is particularly well represented across these supportive and contextual settings. Studies of tango, culturally specific dance forms (72), exercise-based dance, and other structured programs have reported potential psychological benefits across diverse adult and older populations (29, 73). However, the heterogeneity of these populations and intervention formats limits cross-study comparison and disorder-specific inference. Accordingly, these studies broaden the translational and implementation evidence base but should not be weighted equivalently to trials conducted in diagnostically or symptomatically defined depression or anxiety populations.

Across supportive and contextual studies, dance-based interventions have also been associated with functional and psychosocial outcomes, including quality of life, social connectedness, interpersonal functioning, motivation, sleep, physical confidence, and treatment engagement (68, 74–76). These outcomes may be clinically relevant to recovery and participation, particularly when symptoms coexist with social withdrawal or functional impairment. However, improvements in functional or psychosocial outcomes should not be interpreted as direct evidence of efficacy for depressive or anxiety disorders, nor should they be assumed to mediate symptom improvement unless such pathways are tested directly. Future studies should therefore assess these outcomes alongside symptom change and report attendance, dropout, satisfaction, adverse events, and post-intervention continuation of movement or social activity.

Together, these supportive and contextual findings broaden the potential translational relevance of dance-based interventions while remaining distinct from disorder-specific evidence.

### 4.4 Direct comparison between RDI and DMT

RDI and DMT should be regarded as related but clinically distinct approaches rather than interchangeable forms of dance-based treatment (68). RDI is generally more structured and activity-based, with a broader evidence base spanning community, preventive, rehabilitation, adjunctive, and symptom-defined populations (22, 77). DMT, by contrast, is explicitly psychotherapy-oriented and is more frequently studied in clinically symptomatic populations, particularly adults with depression and related internalizing difficulties (9, 10). Their outcome profiles also differ, with RDI studies more commonly emphasizing symptom, functional, and psychosocial outcomes, whereas DMT studies more often include psychotherapy-relevant domains such as emotional expression, body awareness, and interpersonal functioning (68).

The two evidence bases also differ methodologically: RDI research is broader but more heterogeneous, whereas DMT research is more clinically focused but comparatively smaller (68). Accordingly, the greater breadth of the RDI literature should not be interpreted as stronger clinical evidence, because a substantial proportion of RDI studies were conducted in symptom-defined, supportive, or contextual populations rather than diagnostically defined clinical samples. Direct head-to-head comparisons were limited among the studies identified in this review, and these differences should not be interpreted as evidence of relative superiority or as validated treatment-matching rules.

## 5 Candidate mechanisms and measurement priorities

**Table 2** and **Figure 2** summarize candidate mechanisms and feasible measurement targets relevant to dance-based interventions for depression and anxiety. Five broad pathways are considered: rhythmic entrainment and structured attention, behavioral activation and reward engagement, emotion regulation and repetitive negative thinking, interoceptive and autonomic regulation, and social connectedness and interpersonal co-regulation.

**Figure 2:**
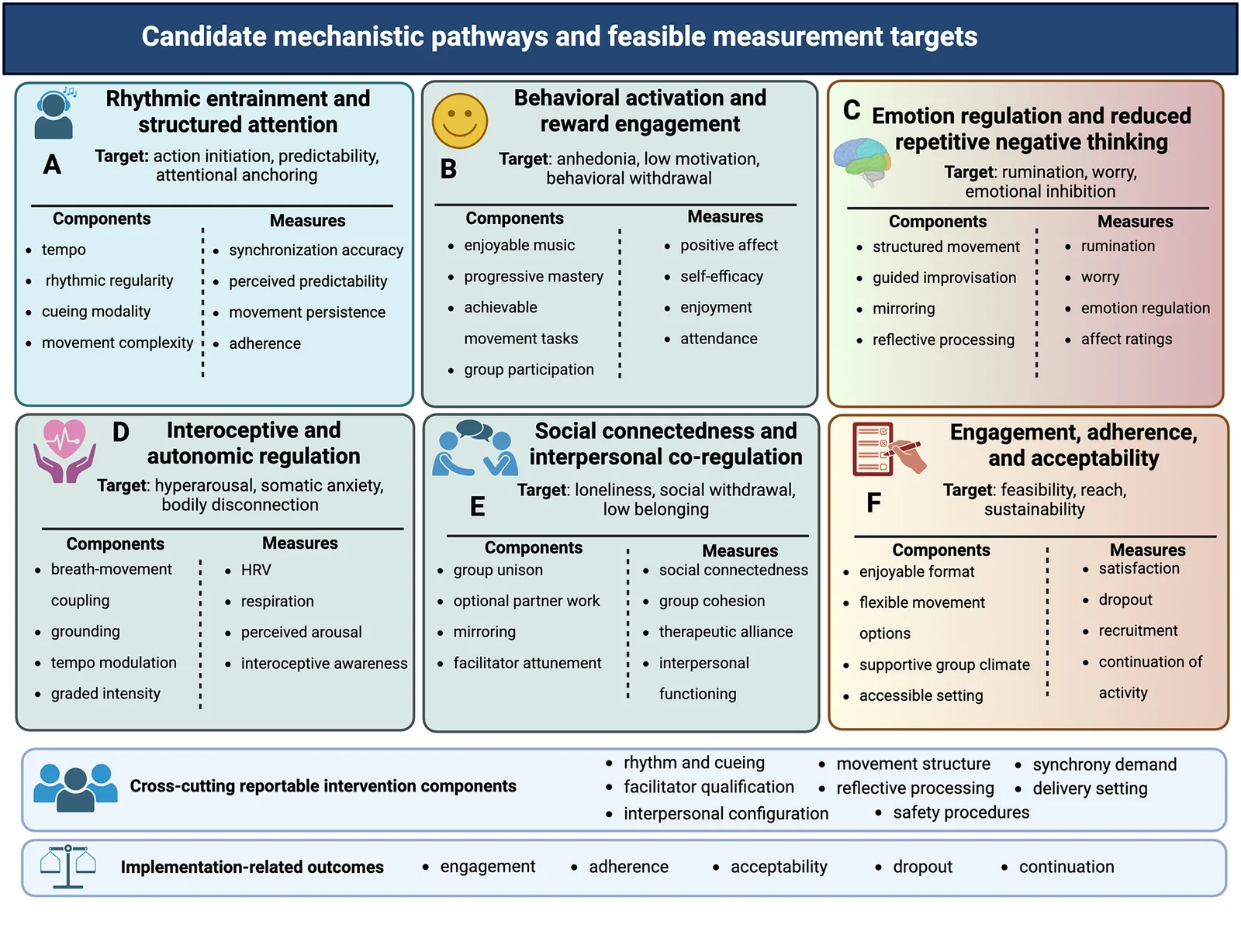
Candidate mechanistic pathways and feasible measurement targets for dance-based mental health interventions. **(A)** Rhythmic entrainment and structured attention; **(B)** behavioral activation and reward engagement; **(C)** emotion regulation and reduced repetitive negative thinking; **(D)** interoceptive and autonomic regulation; **(E)** social connectedness and interpersonal co-regulation; and **(F)** engagement, adherence, and acceptability as an implementation-related domain. For each domain, potentially modifiable intervention components and feasible process or implementation measures are shown. Cross-cutting reportable intervention components and clinical, functional, and implementation outcomes are presented separately. The relationships represented are hypothesized or candidate associations derived from the current evidence base and should not be interpreted as confirmed causal pathways or established mediators of therapeutic change. Psychological, interpersonal, autonomic, or behavioral variables should be described as mediators only when supported by appropriate temporal ordering and mediation or causal pathway analyses.

The evidence supporting these pathways is uneven. For the purposes of this review, direct clinical evidence refers to dance-, RDI-, or DMT-based studies in which candidate process variables were assessed alongside depression-, anxiety-, or related mental health outcomes. Indirect supportive evidence refers to findings from adjacent fields, including rhythm and sensorimotor synchronization research, exercise and reward science, emotion-regulation research, interoception and autonomic physiology, psychotherapy processes, and interpersonal synchrony. Current interpretation refers to the provisional mechanistic inference that can reasonably be drawn from these combined sources. Some processes are informed by direct findings from dance or DMT studies (55, 56, 63, 75), whereas others are supported primarily by adjacent research on rhythm and motor timing (18), exercise and reward processing (78), emotion regulation (79), interoception (80), autonomic physiology (81), psychotherapy processes (82), and interpersonal synchrony (83). Accordingly, the pathways discussed below should be interpreted as candidate mechanisms and measurement targets rather than established mediators of therapeutic change.

Importantly, physiological or neurobiological changes should not be interpreted as evidence of mediation unless supported by appropriate temporal and causal pathway analyses. Concurrent changes in process measures and clinical outcomes indicate association rather than demonstrated mediation.

### 5.1 Mechanistic framework

In this review, candidate mechanisms were considered clinically relevant when they met three criteria. First, they were plausibly linked to depression, anxiety, or transdiagnostic internalizing processes. Second, they might be influenced by reportable and potentially modifiable components of dance-based interventions, such as rhythm-related parameters, movement structure, physical intensity, synchrony demand, guided reflection, interpersonal configuration, or group format. Third, they could be assessed using feasible clinical, behavioral, self-report, interpersonal, or physiological measures. These criteria were used to select and organize the candidate pathways and measurement targets discussed below.

### 5.2 Rhythmic entrainment and structured attention

Rhythm is a defining feature of many dance interventions but remains insufficiently characterized in clinical research. Research on auditory beat perception and sensorimotor synchronization suggested that predictable temporal structure can support movement timing, anticipation, coordination, and sustained action (18, 19, 84, 85). In dance-based interventions, rhythmic structure might also provide an external attentional scaffold that organizes movement and reduces the cognitive demands of initiating and maintaining action (19). Potentially relevant intervention features included tempo, beat salience, rhythmic regularity, cueing modality, movement complexity, and progression (67). Candidate measures included synchronization accuracy, timing variability, movement persistence, perceived predictability, attentional performance, and adherence to movement sequences (86).

Direct dance-based mental health evidence for this pathway remains limited. Few studies have directly tested whether changes in rhythmic synchronization, temporal predictability, or attention account for subsequent improvements in depressive or anxiety-related outcomes (49, 67, 87). Accordingly, rhythmic entrainment and structured attention should currently be regarded as candidate processes and measurement targets rather than validated rhythm-related dose variables.

### 5.3 Behavioral activation and reward engagement

Reduced activity, anhedonia, low motivation, and behavioral withdrawal are common in depression and may also contribute to functional impairment across internalizing conditions (36). Dance-based interventions might influence these processes by combining physical activation with music, enjoyment, novelty, mastery, and opportunities for social participation. The combination of repeated action and potentially rewarding experience may provide a plausible pathway linking participation to changes in activation and motivation (49, 76, 88). Potentially relevant components included enjoyable or culturally meaningful music, achievable movement tasks, progressive skill acquisition, and opportunities for successful participation. Candidate process measures included behavioral activation, positive affect, reward responsiveness, self-efficacy, enjoyment, and willingness to continue participation (54, 55).

Direct dance-based evidence for behavioral activation and reward-related processes is currently sparse. Although some studies have reported improvements in mood, participation, enjoyment, or related outcomes, relatively few have tested whether changes in activation, mastery, enjoyment, or reward responsiveness account for subsequent improvements in depressive or anxiety-related symptoms (60, 89). Indirect support is provided by the broader literature on behavioral activation, physical activity, and reward processing, which supports the relevance of activation and reward-related processes to depressive symptoms and anhedonia (90). Taken together, behavioral activation and reward engagement remain plausible candidate pathways. Reward-related or neurobiological changes should not, however, be interpreted as evidence of dopaminergic mediation unless such pathways are directly measured and formally tested.

### 5.4 Emotion regulation and reduction of repetitive negative thinking

Rumination, worry, emotional avoidance, and reduced flexibility in emotion regulation are clinically relevant processes across depression and anxiety (91). Dance-based interventions may influence these processes by directing attention toward movement, rhythm, body position, interpersonal cues, and present-moment experience. Structured dance can provide an embodied attentional anchor, whereas DMT may additionally use movement exploration and reflective processing to support emotional expression and integration (22). Relevant process measures include rumination, worry, emotion regulation, emotional avoidance, affective change, and patterns of emotional expression or withdrawal. Ecological momentary assessment may also help determine whether changes occurring during or shortly after sessions generalize to everyday emotional functioning (92). Direct evidence that changes in emotion regulation or repetitive negative thinking account for subsequent clinical improvement is currently sparse, although broader emotion-regulation and psychotherapy research provides indirect support for these processes as clinically relevant targets (93, 94). Emotion regulation and repetitive negative thinking therefore remain plausible therapeutic processes requiring direct longitudinal testing. Future studies should distinguish temporary changes in emotional state from sustained changes in regulatory capacity and test whether these changes are associated with depressive symptoms, anxiety-related distress, or functional outcomes.

### 5.5 Interoceptive and autonomic regulation

Anxiety and depression may involve dysregulated arousal, altered bodily awareness, somatic distress, fatigue, sleep disturbance, and heightened sensitivity to bodily sensations (95). Dance-based interventions may influence these processes through breath–movement coupling, grounding, posture, rhythmic predictability, tempo modulation, and graded bodily activation. Candidate measurement targets include heart rate variability, respiration, perceived arousal, somatic anxiety, interoceptive awareness, body awareness, sleep quality, and fatigue. Emerging dance-related studies have begun to examine autonomic measures, but this remains an early area of research (65, 96). Broader research on interoception, autonomic regulation, stress physiology, and physical activity provides indirect support for the relevance of this pathway (80, 97). Importantly, changes in heart rate variability, respiration, or other physiological indices should be interpreted as candidate process markers rather than evidence of mediation unless their role in clinical improvement is tested directly.

### 5.6 Social connectedness and interpersonal co-regulation

Social isolation, loneliness, interpersonal avoidance, and reduced belonging are closely relevant to depression and anxiety (91). Group-based dance and DMT may influence these processes through shared movement, interpersonal synchrony, mirroring, mutual attention, group cohesion, and therapist or facilitator attunement (55). These interpersonal experiences may contribute to affiliation, perceived safety, belonging, and confidence in social participation (98). Candidate measures include loneliness, perceived social connectedness, group cohesion, therapeutic alliance, interpersonal functioning, perceived safety, and qualitative accounts of group experience (14). When relevant to the research question, movement synchrony may also be assessed using video-based analysis, motion tracking, or wearable sensors (99).

Direct evidence that interpersonal synchrony, connectedness, or co-regulation mediates clinical improvement is still insufficient, whereas interpersonal synchrony and psychotherapy-process research provide indirect support for this pathway (38, 75, 86). Objective movement synchrony should therefore not be assumed to represent therapeutic benefit. Future studies should distinguish objective movement synchrony from subjective experiences of affiliation, belonging, safety, and therapeutic connection and examine whether these processes predict subsequent clinical or functional improvement.

### 5.7 Priorities for mechanism-informed trials

Future mechanism-informed trials should assess three linked levels: intervention components, proximal process measures, and clinical or functional outcomes (12). Intervention reporting should capture relevant features such as physical intensity, movement structure, rhythm-related parameters, synchrony demand, interpersonal configuration, facilitator qualification, and reflective processing (38). Process measures should be selected according to a clear theoretical rationale rather than applied as a broad fixed battery. For example, studies manipulating rhythmic predictability should prioritize temporal coordination and attention-related measures, whereas studies focused on interpersonal synchrony should prioritize interpersonal, affiliative, and group-process outcomes (80).

Longitudinal measurement and adequately powered mediation or process analyses are particularly important. Where possible, studies should establish temporal ordering by assessing intervention exposure, proximal process change, and subsequent clinical or functional outcomes at distinct time points. Candidate processes should be described as mediators only when temporal ordering and appropriate mediation or causal pathway analyses support such an inference; otherwise, they should remain candidate mechanisms and measurement targets. This component–process–outcome approach may help determine which intervention features are associated with benefit and through which pathways.

## 6 Translational and implementation framework

### 6.1 From evidence to implementation

The evidence reviewed in Sections 4 and 5 provides a rationale for considering dance-based interventions as adjunctive, preventive, or supportive approaches in mental health and related healthcare contexts (9, 11, 12, 14). However, current evidence does not support a single standardized intervention protocol, validated rhythm-related dosage parameters, or an evidence-based algorithm for matching specific dance formats to particular symptom profiles.

Translation should therefore focus on how interventions are described, who delivers them, where they are delivered, what level of clinical support is available, how safety is managed, and which outcomes are monitored. These considerations are particularly important because dance-based interventions differ substantially in clinical intent, facilitator qualification, therapeutic processing, delivery setting, and responsibility for screening and referral. **Table 3** summarizes practical considerations for intervention reporting, delivery context, facilitator qualification, monitoring, safety, and referral. The taxonomy and FITT + 3R framework proposed in this review are provisional conceptual and reporting tools intended to support research and implementation planning; neither should be interpreted as a validated treatment prescription, dosing framework, or clinical decision rule. Their utility and individual components require prospective empirical evaluation. The implementation considerations outlined below are review-derived and non-prescriptive; they were not developed through a formal guideline, consensus, or clinical practice recommendation process.

### 6.2 FITT + 3R reporting framework

A provisional reporting framework FITT + 3R for dance-based interventions can build on the conventional FITT model of frequency, intensity, time, and type (35). To capture features not adequately represented by FITT alone, we propose three additional reporting dimensions: Rhythm, Relationship, and Reflection. We therefore refer to this review-specific extension as FITT + 3R (**Figure 3**).

**Figure 3:**
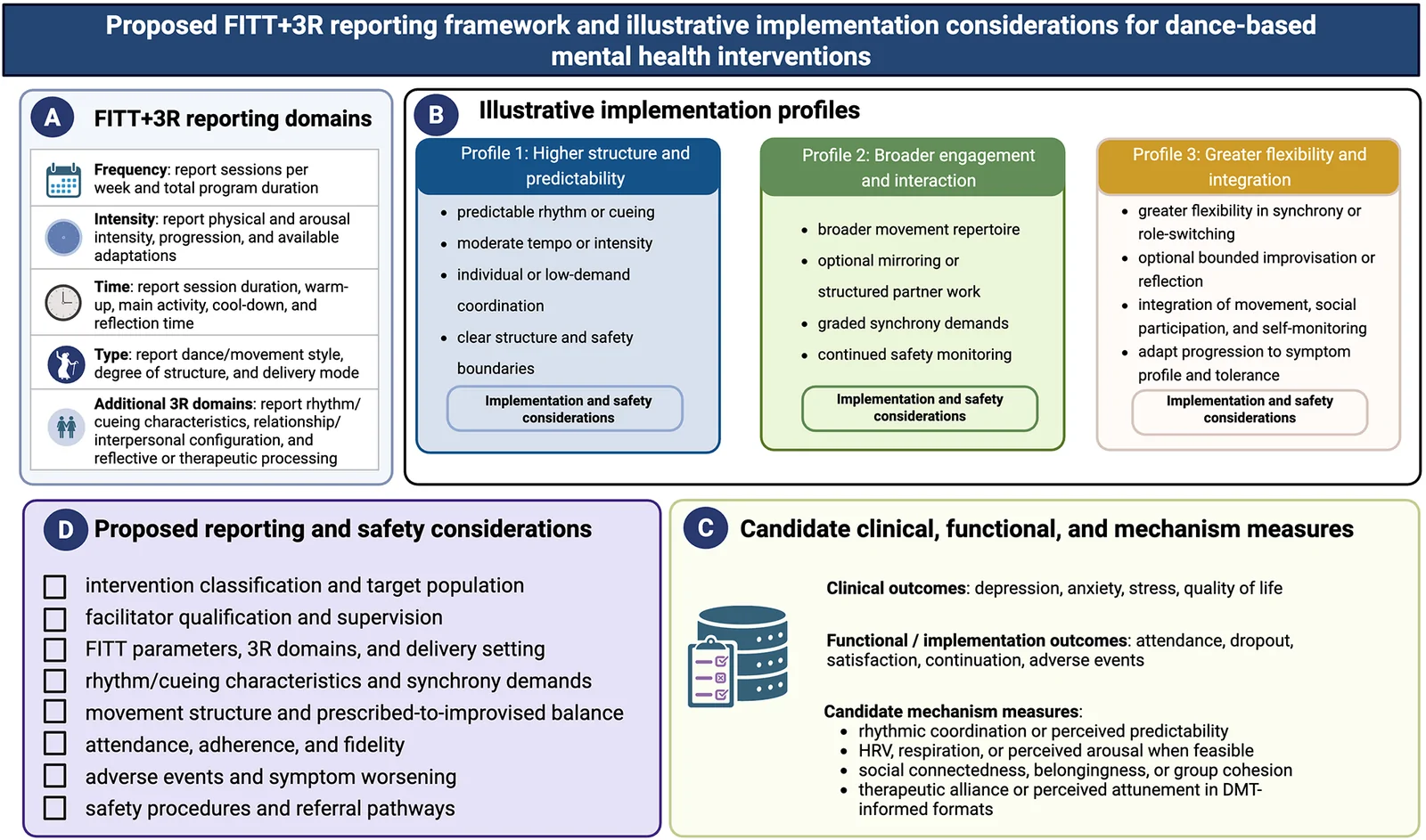
Proposed FITT+3R reporting framework and illustrative implementation considerations for dance-based mental health interventions. Panel **(A)** presents the conventional FITT dimensions—Frequency, Intensity, Time, and Type—together with three additional review-specific reporting domains: Rhythm, Relationship, and Reflection (3R). Panel **(B)** provides illustrative implementation profiles characterized by differing levels of structure, predictability, interpersonal engagement, and flexibility. These profiles are intended as examples for organizing intervention description and planning and do not represent a required treatment sequence, validated progression model, clinical decision pathway, or treatment algorithm. Panel **(C)** summarizes candidate clinical, functional, implementation, and process measures, whereas Panel **(D)** outlines proposed reporting and safety considerations. FITT+3R is a provisional, non-prescriptive conceptual and reporting framework and requires prospective empirical evaluation before it can be used to guide intervention dose, progression, or treatment matching.

Frequency should describe sessions per week and total program duration. Time should specify overall session length and, where relevant, the duration of warm-up, main movement activity, cool-down, and reflective processing. Intensity should describe physical exertion, arousal demands, movement amplitude, and progression. Type should identify the dance or movement approach, degree of structure, facilitator qualification, delivery mode, and intervention classification (12, 35, 37).

Rhythm-related reporting should describe music or cueing source, approximate tempo range when available, rhythmic regularity, cueing modality, movement complexity, and progression (100). Relationship-related reporting should specify whether movement is primarily solo, dyadic, small-group, whole-group, synchronous, mirrored, partner-based, or performance-oriented (101). Reflection-related reporting should indicate whether sessions include no reflection, brief supportive discussion, structured emotional processing, or formal psychotherapeutic integration (102).

FITT+3R is intended to improve intervention description, replication, cross-study comparison, safety planning, and component-level research. Its clinical relevance and the optimal specification of individual dimensions require prospective empirical testing before the framework can be used to guide intervention dose, progression, or treatment matching.

### 6.3 Delivery contexts and levels of clinical support

Dance-based interventions may be delivered across a continuum of community and healthcare settings. The appropriate level of facilitator qualification, clinical governance, monitoring, and referral capacity may vary according to the target population, delivery context, and degree of clinical complexity. Community and preventive programs may be delivered in schools, universities (27), workplaces (55), community centers, or older adult services (71). In these settings, priorities include accessibility, cultural relevance, participant choice, physical safety, and clear procedures for responding to emerging mental health or medical concerns.

Dance-based programs may also be incorporated as adjunctive components within primary care, rehabilitation, psychosocial support, or other healthcare services. Relevant implementation considerations include appropriate screening procedures, clearly defined facilitator roles, routine outcome monitoring, and pathways for clinical review or referral when additional support is needed (103, 104).

DMT and clinically supervised DMT-informed interventions may be integrated into settings requiring greater therapeutic containment or multidisciplinary coordination, including mental health, trauma, psychosomatic, rehabilitation, oncology, and geriatric services. These contexts may warrant appropriately qualified practitioners, clear professional boundaries, clinical supervision, safety monitoring, and coordination with relevant healthcare providers (101).

This continuum describes differences in delivery context and required clinical support rather than a validated hierarchy of treatment effectiveness. DMT and other dance-based interventions should not be regarded as substitutes for indicated psychiatric, psychological, or medical care. Rather, they are best considered complementary or adjunctive approaches that may provide additional benefit when appropriately integrated into multidisciplinary care and coordinated with relevant healthcare professionals.

### 6.4 Safety screening and referral pathways

Relevant safety considerations include both physical and psychological risk. Physical screening considerations may include cardiopulmonary conditions, musculoskeletal limitations, pain, fatigue, mobility restriction, balance or fall risk, pregnancy-related considerations, and medication effects that may influence participation (14, 105). Potential risk-mitigation measures include appropriate warm-up and cool-down, graded intensity, low-impact or seated alternatives when needed, hydration, clear stop rules, and procedures for responding to injury or acute physical distress.

Psychological safety considerations include predictable session structure, informed choice, bodily autonomy, and careful management of touch, visibility, proximity, partner work, improvisation, and emotionally evocative movement (106). Participants should be able to pause, observe, modify activities, or decline specific forms of participation. Trauma-informed principles commonly emphasize safety, choice, collaboration, predictability, and avoidance of coercive participation (107).

Programs may predefine circumstances that prompt clinical assessment, specialist review, or urgent referral. These may include suicidal ideation involving intent or plan, recent self-harm, mania or hypomania, psychosis, severe dissociation, severe trauma-related destabilization, uncontrolled substance use, or rapid clinical deterioration (108). Medical review may also be required when physical activity presents substantial risk because of unstable cardiovascular or respiratory disease, acute injury, uncontrolled pain, high fall risk, or other significant medical concerns.

These recommendations are pragmatic safeguards rather than validated decision rules. They should be adapted to local professional standards, clinical governance, emergency procedures, and referral pathways. DMT and other dance-based interventions should not be regarded as substitutes for indicated psychiatric, psychological, or medical care. Rather, they are best considered complementary or adjunctive approaches that may provide additional benefit when appropriately integrated into multidisciplinary care and coordinated with relevant healthcare professionals.

### 6.5 Monitoring and implementation outcomes

Evaluation may include both clinical and functional outcomes as well as implementation-relevant indicators. Studies and programs are encouraged to report recruitment, reach, attendance, dropout, satisfaction, adherence, perceived barriers, adverse events, and continuation of activity after the formal intervention ends (37, 109). Engagement, adherence, and acceptability may be measured rather than assumed to be inherent advantages of dance-based interventions. Enjoyment, cultural relevance, accessibility, cost, delivery format, facilitator style, and social context may influence participation, but these relationships require empirical evaluation (58, 73).

Adverse-event monitoring may include both physical and psychological events, such as injury, pain exacerbation, dizziness, panic symptoms, dissociation, emotional destabilization, symptom worsening, or referral for urgent assessment. Transparent reporting is necessary for evaluating feasibility and safety and should be incorporated into both clinical trials and real-world implementation studies (42).

Key domains for implementation research include reach, acceptability, cost, equity, cultural fit, facilitator training requirements, fidelity, adaptation, scalability, sustainability, and integration with existing care pathways. These outcomes should be examined across relevant settings, including primary care, community mental health, schools, universities, rehabilitation, older adult services, and online or hybrid delivery (68).

The broader implementation goal is to determine whether promising dance-based interventions can be delivered safely, equitably, feasibly, and sustainably in real-world healthcare and community settings.

## 7 Discussion

### 7.1 Current conclusions and contribution of this review

This review extends previous reviews and meta-analyses by moving beyond the question of whether dance-based interventions are broadly associated with psychological benefit and by addressing several sources of conceptual and evidentiary ambiguity in the field (11, 14, 15, 73). It distinguishes structured RDI from DMT according to clinical intent, delivery characteristics, therapeutic processing, and facilitator roles. It also organizes the clinical literature according to evidentiary directness, distinguishing diagnostically defined disorders from symptom-defined populations, supportive evidence from other clinical populations, and broader contextual evidence. In parallel, direct mechanistic evidence is separated from indirect support derived from adjacent fields and interpreted within a component–process–outcome framework. In addition, this review proposes an operational taxonomy, the provisional FITT + 3R reporting framework, and non-prescriptive implementation considerations to improve intervention description, comparison, and future translational research.

The available evidence supports cautious optimism regarding both RDI and DMT, but the two approaches should not be treated as interchangeable. The most direct psychotherapy-oriented evidence remains concentrated in DMT for adult depression, whereas RDI has a broader but more heterogeneous evidence base spanning depressive and anxiety-related symptoms, psychosocial outcomes, and supportive or contextual populations (9, 15, 16). These findings support continued investigation of RDI and DMT as adjunctive or supportive approaches, but they do not establish comparative superiority, standardized intervention dose, or validated rules for matching particular dance formats to specific symptom profiles.

### 7.2 Remaining uncertainties and limitations

The central uncertainties concern not only whether dance-based interventions can be beneficial, but which components contribute to benefit, through which processes, for whom, and under what delivery conditions. Current evidence does not establish rhythm-related dose–response relationships, reliable patient–intervention matching rules, or causal mediation by the proposed psychological, interpersonal, autonomic, or neurobiological processes. Variability in intervention reporting and study design continues to limit cross-study comparison, while evidence regarding durability, adverse events, implementation, cultural adaptation, accessibility, and equity remains incomplete (49, 101, 110, 111). These gaps limit the precision and generalizability of current conclusions and cannot be resolved simply by conducting larger trials using otherwise similar designs.

This review also has several limitations. It was designed as a structured narrative review and mechanism-informed translational synthesis rather than a systematic review or meta-analysis and therefore does not provide pooled effect estimates, comprehensive risk-of-bias ratings, or formal certainty-of-evidence grading. Because the evidence map was purposively constructed rather than generated through a closed systematic eligibility process, selection bias may have influenced which studies were retained and emphasized. Although structured searching and predefined evidentiary roles improved transparency, study classification and synthesis remained partly interpretive and may also have been influenced by author judgment. Restriction to English-language peer-reviewed literature may also have excluded culturally specific or regionally embedded dance practices and implementation experiences.

In addition, the operational taxonomy, candidate mechanism framework, FITT + 3R framework, and implementation considerations proposed here remain provisional. Existing studies do not always provide sufficient detail for precise intervention classification, and the proposed mechanistic pathways have rarely been tested using longitudinal mediation or causal pathway designs. Similarly, FITT + 3R and the implementation considerations have not undergone formal validation or consensus development. They should therefore be interpreted as conceptual and reporting tools rather than validated treatment prescriptions, dosing algorithms, or clinical decision rules.

### 7.3 Future research priorities

#### 7.3.1 Rigorous efficacy trials

Future research should prioritize better-defined interventions, better-characterized populations, and mechanism-informed trial designs, rather than simply replicating heterogeneous efficacy studies with larger samples. Trials should clearly distinguish DMT from RDI and other dance-based formats, specify whether participants have formally diagnosed disorders or symptom-defined presentations, and use credible active comparators where feasible. Intervention components should be reported in sufficient detail to permit replication and comparison, with the provisional FITT + 3R framework providing one possible structure; however, its dimensions require empirical validation before being used prescriptively.

#### 7.3.2 Mechanism-informed component studies

Mechanism-informed studies should link specific intervention components to theoretically matched proximal process measures and subsequent clinical or functional outcomes. Longitudinal designs should establish temporal ordering, and mediation or causal pathway analyses should be adequately powered before candidate psychological, interpersonal, autonomic, or neurobiological variables are described as mediators. Direct comparisons between RDI and DMT, as well as dismantling or optimization studies manipulating rhythm, movement structure, synchrony, interpersonal configuration, or reflective processing, could help determine whether particular components contribute effects beyond general physical activity, music exposure, social participation, therapeutic attention, or expectancy.

#### 7.3.3 Implementation and safety research

Implementation and safety research should proceed alongside efficacy and mechanism studies. Future work should examine adverse events, acceptability, adherence, equity, cultural fit, facilitator training, fidelity, adaptation, cost, scalability, sustainability, and referral needs across healthcare and community settings. Particular attention should be paid to whether implementation requirements vary across populations, cultures, settings, and levels of clinical complexity. Dance-based interventions should not be presented as substitutes for indicated psychiatric, psychological, or medical care; rather, implementation studies should determine how clearly defined interventions can be delivered safely, feasibly, equitably, and sustainably as adjunctive or supportive approaches.

## 8 Conclusion

RDI and DMT should be distinguished as related but clinically and conceptually different approaches rather than treated as interchangeable forms of dance-based intervention. Current evidence suggests potential benefits for depressive, anxiety-related, and psychosocial outcomes, but the literature remains heterogeneous in populations, intervention formats, and evidentiary directness, limiting firm conclusions regarding comparative efficacy, optimal dose, or treatment matching. The operational taxonomy and provisional FITT + 3R framework proposed in this review may improve intervention description, evidence interpretation, and future research, but both require further empirical testing and validation before they can support prescriptive clinical use.
