# Supplementary material for: Rhythmic dance interventions and dance/movement therapy for depression and anxiety: a structured narrative review of clinical evidence, candidate mechanisms, and implementation considerations
Source: Front Psychiatry. 2026 Oct 19;17:1944858. doi: 10.3389/fpsyt.2026.1944858 (PMC13726093; doi:10.3389/fpsyt.2026.1944858)
Supplement: Supplementary file 1 [file Table1.docx]

Supplementary Table S1. Database-specific search strategies

Searches were updated on 10–11 September 2026. No study-design filter was applied. Three complementary searches were conducted for each database: clinical evidence, candidate mechanisms, and implementation/safety. Search syntax was adapted to each database platform.

| Database/platform | Search domain | Exact search strategy |
| --- | --- | --- |
| PubMed/MEDLINE | Clinical evidence | ("Dance Therapy"[Mesh] OR "dance therapy"[Title/Abstract] OR "dance/movement therapy"[Title/Abstract] OR "dance movement therapy"[Title/Abstract] OR "dance movement psychotherapy"[Title/Abstract] OR "rhythmic dance"[Title/Abstract] OR "structured dance"[Title/Abstract] OR "creative movement"[Title/Abstract] OR "rhythmic movement"[Title/Abstract] OR (danc*[Title/Abstract] AND (intervention*[Title/Abstract] OR therap*[Title/Abstract] OR program*[Title/Abstract] OR treatment*[Title/Abstract])) ) AND ( "Depressive Disorder"[Mesh] OR "Anxiety Disorders"[Mesh] OR depress*[Title/Abstract] OR anxi*[Title/Abstract] OR "major depressive disorder"[Title/Abstract] OR "generalized anxiety disorder"[Title/Abstract] OR "social anxiety"[Title/Abstract] OR stress[Title/Abstract] OR "psychological distress"[Title/Abstract] OR "internalizing symptom*"[Title/Abstract] OR "internalising symptom*"[Title/Abstract] OR "psychological well-being"[Title/Abstract] OR "psychological wellbeing"[Title/Abstract] OR "quality of life"[Title/Abstract] ) |
|  | Candidate mechanisms | ("Dance Therapy"[Mesh] OR "dance therapy"[Title/Abstract] OR "dance/movement therapy"[Title/Abstract] OR"dance movement therapy"[Title/Abstract] OR "dance movement psychotherapy"[Title/Abstract] OR"rhythmic dance"[Title/Abstract] OR "structured dance"[Title/Abstract] OR "creative movement"[Title/Abstract] OR "rhythmic movement"[Title/Abstract] OR(danc*[Title/Abstract] AND (intervention*[Title/Abstract] OR therap*[Title/Abstract] OR program*[Title/Abstract] OR treatment*[Title/Abstract])))AND(rhythm*[Title/Abstract] OR entrain*[Title/Abstract] OR synchron*[Title/Abstract] OR "behavioral activation"[Title/Abstract] OR "behavioral activation"[Title/Abstract] OR reward*[Title/Abstract] OR anhedoni*[Title/Abstract] OR motivation*[Title/Abstract] OR "emotion regulation"[Title/Abstract] OR rumination[Title/Abstract] OR interocept*[Title/Abstract] OR autonomic[Title/Abstract] OR "heart rate variability"[Title/Abstract] OR HRV[Title/Abstract] OR" social connectedness"[Title/Abstract] OR "social connection"[Title/Abstract] OR "interpersonal synchrony"[Title/Abstract] OR "interpersonal co-regulation"[Title/Abstract] OR "interpersonal coregulation"[Title/Abstract] OR "therapeutic alliance"[Title/Abstract] OR attunement[Title/Abstract] OR mirroring[Title/Abstract] ) |
|  | Implementation/safety | ("Dance Therapy"[Mesh] OR "dance therapy"[Title/Abstract] OR "dance/movement therapy"[Title/Abstract] OR dance movement therapy"[Title/Abstract] OR "dance movement psychotherapy"[Title/Abstract] OR "rhythmic dance"[Title/Abstract] OR "structured dance"[Title/Abstract] OR "creative movement"[Title/Abstract] OR "rhythmic movement"[Title/Abstract] OR (danc*[Title/Abstract] AND (intervention*[Title/Abstract] OR therap*[Title/Abstract] OR program*[Title/Abstract] OR treatment*[Title/Abstract])) ) AND (implement*[Title/Abstract] OR feasib*[Title/Abstract] OR acceptab*[Title/Abstract] OR adherence[Title/Abstract] OR fidelity[Title/Abstract] OR safety[Title/Abstract] OR "adverse event*"[Title/Abstract] OR injur*[Title/Abstract] OR dropout[Title/Abstract] OR screening[Title/Abstract] OR referral[Title/Abstract] OR accessib*[Title/Abstract] OR equity[Title/Abstract] OR scalab*[Title/Abstract] OR sustainab*[Title/Abstract] OR facilitator*[Title/Abstract] OR qualification*[Title/Abstract] OR reporting[Title/Abstract] OR "trauma informed"[Title/Abstract] OR "trauma-informed"[Title/Abstract] OR "stepped care"[Title/Abstract] OR "stepped-care"[Title/Abstract] ) |
| Web of Science Core Collection | Clinical evidence | TS=( ("dance therapy" OR "dance/movement therapy" OR "dance movement therapy" OR "dance movement psychotherapy" OR "rhythmic dance" OR "structured dance" OR "creative movement" OR "rhythmic movement" OR (danc* AND (intervention* OR therap* OR program* OR treatment*)) ) AND ( depress* OR anxi* OR "major depressive disorder" OR "generalized anxiety disorder" OR "social anxiety" OR stress OR "psychological distress" OR "internalizing symptom*" OR "internalising symptom*" OR "psychological well-being" OR "psychological wellbeing" OR "quality of life" ) ) |
|  | Candidate mechanisms | TS=(("dance therapy" OR "dance/movement therapy" OR "dance movement therapy" OR "dance movement psychotherapy" OR"rhythmic dance" OR "structured dance" OR "creative movement" OR "rhythmic movement" OR (danc* AND (intervention* OR therap* OR program* OR treatment*)) ) AND ( rhythm* OR entrain* OR synchron* OR "behavioral activation" OR "behavioral activation" OR reward* OR anhedoni* OR motivation* OR "emotion regulation" OR rumination OR interocept* OR autonomic OR "heart rate variability" OR HRV OR "social connectedness" OR "social connection" OR "interpersonal synchrony" OR "interpersonal co-regulation" OR "interpersonal coregulation" OR "therapeutic alliance" OR attunement OR mirroring) ) |
|  | Implementation/safety | TS=(( "dance therapy" OR "dance/movement therapy" OR "dance movement therapy" OR "dance movement psychotherapy" OR"rhythmic dance" OR "structured dance" OR "creative movement" OR "rhythmic movement" OR (danc* AND (intervention* OR therap* OR program* OR treatment*)))AND (implement* OR feasib* OR acceptab* OR adherence OR fidelity OR safety OR "adverse event*" OR injur* OR dropout OR screening OR referral OR accessib* OR equity OR scalab* OR sustainab* OR facilitator* OR qualification* OR reporting OR "trauma informed" OR "trauma-informed" OR "stepped care" OR "stepped-care")) |
| Scopus | Clinical evidence | TITLE-ABS-KEY( ("dance therapy" OR "dance/movement therapy" OR "dance movement therapy" OR "dance movement psychotherapy" OR "rhythmic dance" OR "structured dance" OR "creative movement" OR "rhythmic movement" OR (danc* AND (intervention* OR therap* OR program* OR treatment*)) ) AND ( depress* OR anxi* OR "major depressive disorder" OR "generalized anxiety disorder" OR "social anxiety" OR stress OR "psychological distress" OR "internalizing symptom*" OR "internalising symptom*" OR "psychological well-being" OR "psychological wellbeing" OR "quality of life" )) |
|  | Candidate mechanisms | TITLE-ABS-KEY(("dance therapy" OR "dance/movement therapy" OR "dance movement therapy" OR "dance movement psychotherapy" OR "rhythmic dance" OR "structured dance" OR "creative movement" OR "rhythmic movement" OR (danc* AND (intervention* OR therap* OR program* OR treatment*)) ) AND ( rhythm* OR entrain* OR synchron* OR "behavioral activation" OR "behavioural activation" OR reward* OR anhedoni* OR motivation* OR "emotion regulation" OR rumination OR interocept* OR autonomic OR "heart rate variability" OR HRV OR "social connectedness" OR "social connection" OR "interpersonal synchrony" OR "interpersonal co-regulation" OR "interpersonal coregulation" OR "therapeutic alliance" OR attunement OR mirroring ) ) |
|  | Implementation/safety | TITLE-ABS-KEY( ("dance therapy" OR "dance/movement therapy" OR "dance movement therapy" OR "dance movement psychotherapy" OR "rhythmic dance" OR "structured dance" OR "creative movement" OR "rhythmic movement" OR (danc* AND (intervention* OR therap* OR program* OR treatment*)) )AND (implement* OR feasib* OR acceptab* OR adherence OR fidelity OR safety OR "adverse event*" OR injur* OR dropout OR screening OR referral OR accessib* OR equity OR scalab* OR sustainab* OR facilitator* OR qualification* OR reporting OR "trauma informed" OR "trauma-informed" OR "stepped care" OR "stepped-care" )) |
| APA PsycINFO via PsycNet | Clinical evidence | APA PsycNet Advanced Search; database = PsycINFO; field = Any Field: ("dance therapy" OR "dance/movement therapy" OR "dance movement therapy" OR "dance movement psychotherapy" OR "rhythmic dance" OR "structured dance" OR "creative movement" OR "rhythmic movement" OR (danc* AND (intervention* OR therap* OR program* OR treatment*))) AND ( depress* OR anxi* OR "major depressive disorder" OR "generalized anxiety disorder" OR "social anxiety" OR stress OR "psychological distress" OR "internalizing symptom*" OR "internalising symptom*" OR "psychological well-being" OR "psychological wellbeing" OR "quality of life" ) |
|  | Candidate mechanisms | APA PsycNet Advanced Search; database = PsycINFO; field = Any Field: ( "dance therapy" OR "dance/movement therapy" OR "dance movement therapy" OR "dance movement psychotherapy" OR "rhythmic dance" OR "structured dance" OR "creative movement" OR "rhythmic movement" OR (danc* AND (intervention* OR therap* OR program* OR treatment*)) ) AND (rhythm* OR entrain* OR synchron* OR "behavioral activation" OR "behavioural activation" OR reward* OR anhedoni* OR motivation* OR "emotion regulation" OR rumination OR interocept* OR autonomic OR "heart rate variability" OR HRV OR "social connectedness" OR "social connection" OR "interpersonal synchrony" OR "interpersonal co-regulation" OR "interpersonal coregulation" OR "therapeutic alliance" OR attunement OR mirroring ) |
|  | Implementation/safety | APA PsycNet Advanced Search; database = PsycINFO; field = Any Field: ( "dance therapy" OR "dance/movement therapy" OR "dance movement therapy" OR "dance movement psychotherapy" OR "rhythmic dance" OR "structured dance" OR "creative movement" OR "rhythmic movement" OR (danc* AND (intervention* OR therap* OR program* OR treatment*)) ) AND (implement* OR feasib* OR acceptab* OR adherence OR fidelity OR safety OR "adverse event*" OR injur* OR dropout OR screening OR referral OR accessib* OR equity OR scalab* OR sustainab* OR facilitator* OR qualification* OR reporting OR "trauma informed" OR "trauma-informed" OR "stepped care" OR "stepped-care" ) |
| Cochrane Library | Clinical evidence | #1 "dance therapy" OR "dance movement therapy" OR "dance movement psychotherapy" OR "rhythmic dance" OR "structured dance" OR "creative movement" OR "rhythmic movement" OR (danc* NEAR/3 (intervention* OR therap* OR program* OR treatment*))  #2 depress* OR anxi* OR "major depressive disorder" OR "generalized anxiety disorder" OR "social anxiety" OR stress OR "psychological distress" OR (internalizing NEXT symptom*) OR (internalising NEXT symptom*) OR (psychological NEXT wellbeing) OR "quality of life"  #3 #1 AND #2 |
|  | Candidate mechanisms | #1 "dance therapy" OR "dance movement therapy" OR "dance movement psychotherapy" OR "rhythmic dance" OR "structured dance" OR "creative movement" OR "rhythmic movement" OR (danc* NEAR/3 (intervention* OR therap* OR program* OR treatment*))  #4 rhythm* OR entrain* OR synchron* OR "behavioral activation" OR "behavioural activation" OR reward* OR anhedoni* OR motivation* OR "emotion regulation" OR rumination OR interocept* OR autonomic OR "heart rate variability" OR HRV OR "social connectedness" OR "social connection" OR "interpersonal synchrony" OR "interpersonal co-regulation" OR "interpersonal coregulation" OR "therapeutic alliance" OR attunement OR mirroring  #5 #1 AND #4 |
|  | Implementation/safety | #1 "dance therapy" OR "dance movement therapy" OR "dance movement psychotherapy" OR "rhythmic dance" OR "structured dance" OR "creative movement" OR "rhythmic movement" OR (danc* NEAR/3 (intervention* OR therap* OR program* OR treatment*))  #6 implement* OR feasib* OR acceptab* OR adherence OR fidelity OR safety OR "adverse event" OR "adverse events" OR injury OR dropout OR screening OR referral OR accessib* OR equity OR scalab* OR sustainab* OR facilitator* OR qualification* OR reporting OR "trauma informed" OR "stepped care"  #7 #1 AND #6 |

The three search domains were intentionally run separately so that clinical-effect studies were not required to contain mechanistic or implementation terms. Search yields are reported in the Methods and Figure S1. For the Cochrane Library, search yields were retained, but full CENTRAL records could not be batch exported under the available institutional access and were therefore not incorporated into automated cross-database deduplication.
